# Exposure to Food and Beverage Advertising on Television among Canadian Adolescents, 2011 to 2016

**DOI:** 10.3390/nu12020428

**Published:** 2020-02-07

**Authors:** Christine D Czoli, Elise Pauzé, Monique Potvin Kent

**Affiliations:** 1 School of Epidemiology and Public Health, University of Ottawa, Ottawa, ON K1G 5Z3, Canada; christine.czoli@gmail.com (C.D.C.); epauz022@uottawa.ca (E.P.); 2 Heart and Stroke Foundation, Ottawa, ON K1Z 8R9, Canada

**Keywords:** food advertising, adolescents, public health, sugary drinks, policy, obesity

## Abstract

Adolescents represent a key audience for food advertisers, however there is little evidence of adolescent exposure to food marketing in Canada. This study examined trends in Canadian adolescents’ exposure to food advertising on television. To do so, data on 19 food categories were licensed from Nielsen Media Research for May 2011, 2013, and 2016 for the broadcasting market of Toronto, Canada. The average number of advertisements viewed by adolescents aged 12–17 years on 31 television stations during the month of May each year was estimated using television ratings data. Findings revealed that between May 2011 and May 2016, the total number of food advertisements aired on all television stations increased by 4%, while adolescents’ average exposure to food advertising decreased by 31%, going from 221 ads in May 2011 to 154 in May 2016. In May 2016, the advertising of fast food and sugary drinks dominated, relative to other categories, accounting for 42% and 11% of all exposures, respectively. The findings demonstrate a declining trend in exposure to television food advertising among Canadian adolescents, which may be due to shifts in media consumption. These data may serve as a benchmark for monitoring and evaluating future food marketing policies in Canada.

## 1. Introduction

The advertising of unhealthy foods and beverages influences the dietary behavior of children and youth and is a likely contributor to obesity and diet related non-communicable diseases [1]. Several comprehensive reviews of the literature have concluded that commercial food advertising is associated with childhood obesity, influencing the food preferences of children, consumption patterns, and purchase requests [1,2,3,4,5,6]. While limited, there is some evidence linking adolescents’ television advertising exposure to their food choices and consumption patterns [1,7], as well as adiposity [1]. Thus, public health experts and policymakers have expressed concerns about food marketing aimed at adolescents [8,9].

In Canada, the prevalence of obesity has increased dramatically since the late 1970s [10]. While this trend appears to have stabilized in the last decade [11], 34% of Canadian youth aged 12–17 years have excess weight or obesity and their diets remain poor [12]. For instance, research has shown that large proportions of Canadian youth do not meet national dietary guidelines [13,14,15]. Furthermore, Canadian adolescents (aged 14–18 years) obtain approximately 55% of their daily energy intake from ultra-processed foods (i.e., manufactured food products containing many ingredients extracted from foods or synthesized as well as additives and few whole foods or typical culinary ingredients) which usually contain excessive amounts of sugar, fat and sodium [16]. Of particular concern is youth consumption of beverages with free sugars (“sugary drinks”), which provide energy, but no nutritional value [17]. In 2004, 17% and 16% of daily calories among Canadian adolescent boys and girls, respectively, were derived from sweetened drinks (including regular soft drinks and fruit drinks), milk and fruit juice [18]. Taken together, these dietary patterns are problematic due to the high intake of energy-dense yet nutrient-poor foods, as well as the associated displacement of more nutritious foods, such as fruits, vegetables, and legumes [16].

Adolescents represent a key target audience for advertisers of foods and beverages [19]. Canadian youth are significant consumers of media, with adolescents aged 12–17 years watching an average of 16.4 h of television per week [20]. In addition, adolescence marks a transition in which children become direct consumers, acquiring the ability to purchase goods with their own money [8]. In the United States (US), marketing expenditures directed at adolescents exceed $1 billion annually [21]. Despite declines in food marketing expenditures for television over time, television remains the dominant medium for such expenditures [22]. Furthermore, as part of integrated marketing campaigns, television advertising is used concurrently with other forms of marketing to maximize the overall impact of ad campaigns on consumer behavior [23,24]. It is therefore important to monitor food advertising on television. This is also critical for informing policies aimed at protecting adolescents from unhealthy food advertising.

In the province of Quebec, commercial advertising is restricted to children under 13 years old, while in the rest of the country, unhealthy food advertising to children under 12 years old is self-regulated by the food and beverage industry [25,26]. Recently, the Government of Canada came close to adopting a law restricting unhealthy food marketing to children [27]. This law, however, like the existing law in Quebec, would not have protected adolescents aged 13 and over [28]. To our knowledge, there is little research examining food and beverage marketing exposure among Canadian adolescents [29]. This represents a critical evidence gap, given that such data are needed to consider marketing restrictions to adolescents, which are increasingly acknowledged as uniquely vulnerable to marketing [30,31]. To fill this gap, the current study sought to examine Canadian adolescents’ exposure to food and beverage advertising on television over time. Given the significant contribution of sugary drinks to the caloric intake of Canadian adolescents, and its documented contribution to weight gain [32,33], an in-depth examination of sugary drink advertising was also conducted. 

## 2. Materials and Methods

Data for food and beverage television advertisements that aired in May 2011, 2013, and 2016 on 34 television stations were licensed from Nielsen Media Research (a marketing research company). As national advertising ratings data are not available in Canada, data were licensed for the Toronto Extended Market (i.e., the city of Toronto and adjacent urban areas all located in the province of Ontario), as it constitutes the largest broadcast market in the country. The month of May was chosen to avoid major holidays that could influence advertising budgets and usual exposure. For each month, data for four weeks of 24 h television programming were analyzed (1 May to 28 May in 2011, 29 April to 26 May in 2013, and 1 May to 28 May in 2016), and included advertising data for 19 food/beverage categories defined by Nielsen, which are known to be frequently advertised to young people [21,34] (Table 1). The three years examined were selected as this study constitutes a secondary analysis of data used for previously funded research [35,36].

### 2.1. Analysis

Nielsen data includes information for all advertisements aired and viewed by an audience for a specified time period. Television viewership data are collected among a stratified probability sample of households that is proportional to the population in which each member, whose demographic characteristics are known, wears a portable device that records when and what they are watching on television [37]. Data are weighted based on age, sex, household size, geography and other characteristics, and estimates of audience viewership at the television extended market level are calculated by Nielsen, and can be examined by specific demographic groups, such as adolescents aged 12 to 17 years. Nielsen data expresses advertising exposure in terms of a “rating”, representing the estimated percentage of the population (or specific demographic group) that viewed a specific advertisement. As has been done previously [38,39], ratings for all advertisements viewed by adolescents were summed for each food/beverage category. Rating sums were then divided by 100 to determine the average number of advertisements seen by individual adolescents. An in-depth analysis was conducted to examine adolescents’ exposure to advertising for beverages with free sugars, given its contribution to the caloric intake of Canadian youth and associated public health concern [18]. While “sugary drinks” contain a broad range of beverage categories [40], the current analysis was limited to the following beverage categories defined by Nielsen: juices, regular soft drinks, sports drinks, and energy drinks.

Of the 34 stations available in 2011, 3 were excluded from the study because Nielsen ceased to record these stations during the examined period. Adolescents’ exposure to food and beverage advertising on the remaining 31 television stations was assessed for each time point (May 2011, 2013, and 2016), overall and by food category. Television stations included in the analysis are listed in Supplementary Table S1. Food/beverage advertising exposure was also examined by television station type, including children specialty stations (Teletoon and YTV), adolescent specialty stations (Much Music and MTV), and generalist stations (all others). Teletoon and YTV were considered child specialty stations as their programming is predominantly intended for children under 13 years. Adolescent specialty stations were classified as such because a large share of their programming focused on either popular music and comedy shows that appeal to youth (i.e., Much; e.g., Playlist, Simpsons, Everybody Hates Chris, Tosh.0) or adolescent-targeted sitcoms and reality TV shows (i.e., MTV; e.g., Degrassi, Student Bodies, 16 and Pregnant, Teen Mom). The number of food advertisements that aired on all stations and adolescents’ exposure were described using frequencies and the percent changes between May 2011 and May 2016 were calculated. Nielsen Spotwatch software was used to extract television advertising exposure data among adolescents and analyses were conducted using IBM SPSS v.25.

### 2.2. Ethical Approval

As this study used licensed media data, ethical approval was not required.

## 3. Results

### 3.1. Food and Beverage Advertisements Aired on Television 

A total of 84,700, 82,432, and 87,825 advertisements for foods and beverages aired on all television stations in May 2011, 2013, and 2016, respectively. Between May 2011 and 2016, the total number of food and beverage advertisements aired increased by 4% (see Table 2). With respect to frequency of advertising, an average of 97.6 and 101.2 advertisements aired per station per day in 2011 and 2016, respectively. The largest increases in the number of food and beverage advertisements aired were for cakes (+713%), candy (+345%), regular soft drinks (+254%), snack foods (+160%), and sports drinks (+179%). The five food/beverage categories that were most heavily advertised in May 2011 included fast food (34% of total ads), chocolate (12%), yogurt (9%), cereal (9%), and cheese (7%). In May 2016, these were fast food (41% of total ads), chocolate (8%), restaurants (7%), snack foods (6%), and cheese (6%) (May 2016).

### 3.2. Adolescents’ Exposure to Food and Beverage Advertising on Television

On average, adolescents were exposed to 221, 180 and 154 food and beverage advertisements in May 2011, 2013 and 2016, respectively. Between May 2011 and 2016, adolescents’ exposure to food and beverage advertising decreased by 31%. As shown in Table 3, this trend differed by food/beverage category: despite decreases in exposure for several categories, large increases were observed for cakes (+340%), candy (+193%), sports drinks (+155%), and regular soft drinks (+111%). The five food/beverage categories to which adolescents were most exposed in May 2011 included fast food (34% of total exposure), cereal (10%), chocolate (10%), yogurt (9%), and cheese (8%). In May 2016, these categories were fast food (42% of total exposure), cheese (7%), cereal (7%), candy (6%), and juices (5%).

Changes in adolescents’ exposure to food and beverage advertising also differed by television station type. For instance, in May 2016, 75% of adolescents’ total exposure was derived from generalist stations (i.e., stations not specifically targeted at children or adolescents), while 24% and 1% were derived from children’s specialty and teen specialty stations, respectively. While adolescents’ exposure to food advertising showed little change between May 2011 and May 2016 on children’s specialty stations (−7%), the magnitude of change in exposure on teen specialty stations (−76%) and generalist stations (−34%) was considerably greater (Table 4).

### 3.3. Beverages with Free Sugars

**Figure 1:**
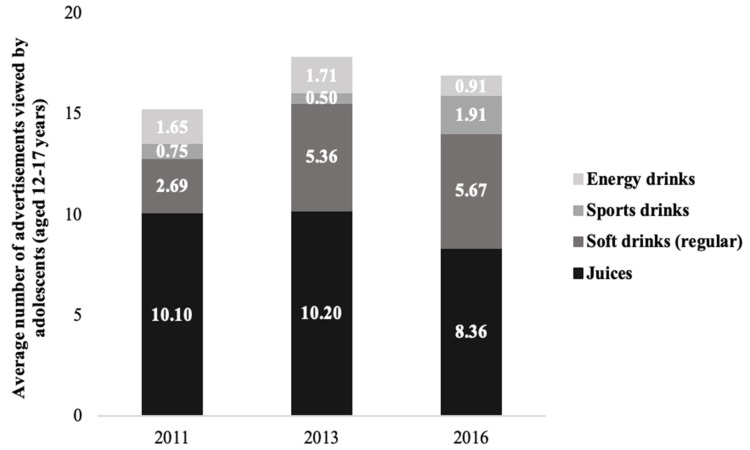
Adolescent exposure to television advertisements for beverages with free sugars, May 2011, 2013 and 2016. Source: Nielsen Media Research, May 2011, 2013, 2016.

When juices, regular soft drinks, sports drinks and energy drinks were considered together, the total number of television advertisements for beverages with free sugars increased by 88% between May 2011 (*n* = 5815) and May 2016 (*n* =10,955). In addition, adolescents’ exposure to advertisements for beverages with free sugars increased by 11% between May 2011 (*n* = 15.19) and May 2016 (*n* = 16.85). However, exposure in May 2016 (*n* = 16.85) was lower than in May 2013 (*n* = 17.52). As shown in Figure 1, decreases in exposure were observed for juices (−17%) and energy drinks (−45%), while increases were observed for regular soft drinks (+111%) and sports drinks (+155%). In 2016, beverages with free sugars represented the second-most heavily advertised food/beverage category among those examined in May 2016, increasing in relative importance from May 2011 (seventh-most heavily advertised category), and May 2013 (third-most heavily advertised category). Correspondingly, beverages with free sugars represented the second-highest levels of exposure among adolescents in May 2016 (11% of total exposure), following that for fast food, increasing in relative importance from 2011 (ranked sixth with respect to exposure) and 2013 (ranked third with respect to exposure). 

## 4. Discussion

The study findings indicate a decline in exposure to food/beverage advertising on television between May 2011 and May 2016 among Canadian youth. This decline was observed despite relatively little change in the total number of food/beverage advertisements aired over the study period. Given that exposure to television advertising is a function of time spent watching television and the frequency of aired advertising, the findings suggest that the observed change may be due to changes in media preferences and consumption among adolescents. Indeed, according to the Canadian Radio-television and Telecommunications Commission (CRTC), Canadians’ weekly viewing of television has decreased from 2011/12 to 2015/16, with the largest decrease among teens aged 12–17 years [20]. Trends in food-related advertising expenditures, given recent shifts in expenditures that favor digital marketing via websites, social media, and mobile applications, also appear to support this interpretation [22]. Despite the observed decline, our findings show that Canadian adolescents are still exposed to more than 150 food and beverage television advertisements per four-week period, which may result in annual exposures of 1800 on television alone. As such, broadcast television remains an important source of food advertising exposure among Canadian adolescents. Its continued monitoring is therefore warranted.

Trends over time in commercial food advertising exposure among adolescents differed by specific food/beverage categories. While some foods and beverages, such as cakes and sports drinks, showed considerable growth over time (+340% and +155%, respectively), they contributed little to adolescents’ total exposure to food/beverage advertising (<1 advertisement exposure in May 2016, respectively). In contrast, categories such as regular soft drinks showed growth over time (+111%), while also contributing more to exposure among youth (6 advertisement exposures in May 2016, up from 3 in May 2011). Finally, while advertising exposure to cereal and cheese decreased over the time period examined (−55% and −38%, respectively), they remained within the five food/beverage categories to which adolescents were most exposed. These findings highlight the importance of regular monitoring of trends in commercial food advertising with respect to both changes within food/beverage categories over time as well as their relative contribution to total exposure. Continued monitoring may also yield greater insights into the variation of commercial food advertising on television and inform the interpretation of observed trends in exposure over time. 

Advertising for fast food dominated relative to other categories, remaining by far the top category with respect to the quantity of television advertising as well as exposure among adolescents over the examined period. Specifically, in May 2016, advertisements for fast food accounted for almost half (42%) of all commercial food advertising exposure among adolescents, as examined in this study. These findings are consistent with analyses examining television advertising exposure among adolescents in the US [8,38]. Findings from the current study also show an increase over time in youth advertising exposure to beverages containing free sugars. When considered together, these sugary drinks represented a significant food/beverage category, with marked increases over time in the quantity of television advertising, as well as in levels of exposure among adolescents, second only to fast food. These findings contrast with declining trends in children’s advertising exposure on television to sugary drinks in both Canada and the US in recent years [41,42,43] and may mark a shift in targeted advertising of older demographic groups. Increased advertising exposure to sugary drinks is concerning, given that consumption of these beverages has been associated with weight gain [32,33] and is a risk factor for various chronic diseases [40]. The dominance of adolescents’ advertising exposure to both fast food and sugary drinks is consistent with elements of the current marketplace. First, it reflects commercial food and beverage marketing expenditures: of the more than $1 billion spent annually on marketing directed at adolescents in the US [21], approximately two-thirds of this is for fast food and sugary drinks [22]. Second, the findings are consistent with this subpopulation’s consumer profile: in addition to influencing family purchases [1], adolescents are direct consumers with access to their own money, and items like fast food and beverages are within their purchasing power [8]. 

Findings from the current study have several policy implications. First, our study revealed that Canadian adolescents are exposed to a considerable volume of food and beverage advertisements, including sugary drinks, on broadcast television. As such, policymakers should consider protecting adolescents when crafting laws restricting unhealthy food marketing directed to children. Given that most (75%) of adolescents’ exposure occurred on generalist stations, statutory restrictions should also apply to programs watched by large numbers of children and adolescents, regardless of the intended audience of a given station or program [24]. As it has been done for children under 13 years of age [9], additional research is needed to understand how various food advertising restrictions on broadcast television may impact adolescents’ exposure. In the United Kingdom, for instance, the restriction of unhealthy food and beverage advertising before 9 pm has been proposed as an alternative to current restrictions based on television audience measurements [44]. However, research on time-based restrictions of alcohol advertising suggests that this may in fact lead to higher advertising exposure among youth [45]. As such, more research is needed to inform policy in this area. 

Second, concerns have been raised that restrictions on unhealthy food and beverage marketing to children may result in unintended consequences in the form of increased marketing targeting adolescents. While such changes have been observed in the US [38], it is not clear whether they may follow in the Canadian context if statutory restrictions exclusively targeting children under 13 years were to be adopted. Continued monitoring of food and beverage marketing to both children and adolescents is warranted. Finally, given that adolescents consumption of broadcast television is declining [20], future research or monitoring should also examine youth’s exposure to food advertising during television or video content viewed on YouTube, online streaming platforms and on smart televisions whose connection to the internet allow advertisers to use meta-data and advanced analytics to target specific demographic groups [46]. While no such study has yet to be conducted, food and beverage advertising during television content viewed online is likely to mirror the predominantly unhealthy nature of food advertising on broadcast television and other media [29,38,39]. Given the increased viewing of television content online, statutory restrictions of unhealthy food and beverage advertising to children and youth should apply to these digital platforms.

To our knowledge, the current study is the first to examine trends over time in food and beverage advertising and exposure among Canadian adolescents. A significant strength of the study is its use of objective exposure data, rather than data based on potential exposure or self-reported measures. The study findings likely underestimate adolescents’ exposure to food and beverage advertising on television, given that not all food and beverage categories were included in the study and exposure to food marketing embedded within television programs (e.g., product placements and sponsorship of broadcasted sporting events) are not captured in the analyzed data [47,48]. Adolescents’ exposure to sugary drink advertising, particularly soft drinks, may also be underestimated as these products are often featured within restaurant advertising because of exclusive marketing agreements that exist between restaurant chains and large beverage manufacturers [49]. Furthermore, some television stations were excluded from the analysis to ensure comparability for data collected across different time periods. For example, TSN, a popular sports station, was excluded because data was not collected by Nielsen for May 2016. However, data collected for TSN in May 2011 suggests that adolescents were exposed to considerable advertising for various food and beverages, and in particular, for sports drinks. Since television ratings data are only available in aggregate, statistical testing could not be conducted to determine whether differences in exposure between May 2011 and May 2016 were statistically significant. The study is also limited by its lack of nutritional analysis of advertised foods and beverages, which would have objectively determined their nutritional quality. However, nearly all the examined food and beverage categories represented largely “unhealthy” foods. Indeed, this bias in food marketing is reflected in youth-targeted marketing expenditures, which are dominated by fast food restaurants, beverages, breakfast cereals, and snack foods, with fruits and vegetables accounting for less than 1% of expenditures [22]. Finally, data were analyzed for the broadcast market of Toronto for the month of May in each examined year; thus, the extent to which the findings represent trends over the entire calendar year and markets across Canada is unclear. Given seasonal variations in advertising, with some products being advertised for only parts of the year, recorded changes in advertising by food category may not be representative of annual trends.

## 5. Conclusions 

The current study demonstrates a declining trend over time in exposure to food and beverage television advertising among Canadian adolescents. This decline was observed despite no considerable changes in the quantity of commercial food advertisements on television, suggesting that this trend may be due to shifts in adolescents’ media preferences and consumption. Adolescents’ commercial food advertising on television is dominated by advertisements for fast food and increasingly, for beverages with free sugars. These data highlight the need to include adolescents in statutory food marketing restrictions in Canada. They may serve as a benchmark for monitoring and evaluating future food and beverage marketing policies in the country. 

## Figures and Tables

**Table 1:** Definitions of Nielsen food/beverage categories included in the study.

Food/Beverage Category	Definition
Cakes	All cakes and puddings, including items that are ready to eat or require additional preparation (excludes frozen pastry and pie shells)
Candy	Confectionary made from sugar, water, flavoring and food coloring (excludes candy with chocolate)
Cereal	Ready-to-eat products marketed as breakfast food (excludes infant cereals and oatmeal)
Cheese	Cheese products in various formats, e.g., brick, string or slice (excludes cottage cheese)
Chocolate	Individually wrapped chocolate and candy bars (excludes boxed chocolate and candy with chocolate)
Compartment snacks	Pre-packaged products comprise of two or more ingredients in separate compartments sold as portable snacks or meals
Cookies	Small baked sweet biscuits
Ice cream	Includes ice cream, frozen yogurt, sherbet, sorbet and frozen treats made from these foods
Pizza	Pizza not sold in restaurants
Portable snacks	Cereal, protein or fruit bars and squares, and fruit snacks
Snack foods	Savory snacks such as chips, pretzels, cheese puffs, and meat-based snacks like jerky (excludes crackers)
Fast food restaurants	Foods sold at restaurants where ordering is conducted at a counter or drive-through, where menu boards are placed above the counter, and the table is cleaned up by the customer
Restaurants	Restaurants that serve prepared food and beverages that are ordered from a menu once seated and are consumed on the premises
Yogurt	Yogurt in tub, tube, and drink form (excludes frozen yogurt)
Juices, drinks and nectars	Sweetened and unsweetened juices and beverages that come in liquid, frozen, concentrated, and powdered forms (excludes water, milk and alternatives, tea and coffee drinks, cocktail mixers, and alcoholic beverages)
Energy drinks	Drink products that are primarily consumed for the purpose of boosting one’s mental and physical stimulation
Soft drinks (regular)	Any non-alcoholic carbonated drink
Soft drinks (diet)	Diet versions of soft drinks
Sports drinks	Drink products that are primarily consumed to rehydrate the body and replace electrolytes lost during physical activity

**Table 2:** Number of television food and beverage advertisements aired, in May 2011, 2013 and 2016.

Food/Beverage Category	Total Advertisements Aired			
	May 2011 *n* (%)	May 2013 *n* (%)	May 2016 *n* (%)	% Change in Ad Frequency May 2011 to 2016
Cakes	52 (0.1)	3 (<0.1)	423 (0.5)	+713%
Candy	814 (1.0)	4893 (5.9)	3623 (4.1)	+345%
Cereal	7349 (8.7)	3044 (3.7)	2992 (3.4)	−59%
Cheese	5931 (7.0)	3731 (4.5)	4924 (5.6)	−17%
Chocolate	10,001 (11.8)	12,165 (14.8)	7140 (8.1)	−29%
Compartment snacks	-	352 (0.4)	1 (<0.1)	-
Cookies	3875 (4.6)	2333 (2.8)	1597 (1.8)	−59%
Ice cream	1318 (1.6)	2387 (2.9)	2626 (3.0)	+99%
Pizza	2332 (2.8)	1258 (1.5)	1498 (1.7)	−36%
Portable snacks	2397 (2.8)	1892 (2.3)	1739 (2.0)	−27%
Snack foods	2158 (2.5)	4850 (5.9)	5609 (6.4)	+160%
Fast food	28,508 (33.7)	26,861 (32.6)	35,652 (40.6)	+25%
Restaurants	5686 (6.7)	4506 (5.5)	5918 (6.7)	+4%
Yogurt	7660 (9.0)	4777 (5.8)	2810 (3.2)	−63%
Juices	3491 (4.1)	4615 (5.6)	4845 (5.5)	+39%
Energy drinks	765 (0.9)	804 (1.0)	857 (1.0)	+12%
Soft drinks (regular)	1199 (1.4)	3878 (4.7)	4247 (4.8)	+254%
Soft drinks (diet)	804 (0.9)	-	318 (0.4)	−60%
Sports drinks	360 (0.4)	83 (0.1)	1006 (1.1)	+179%
TOTAL	84,700 (100)	82,432 (100)	87,825 (100)	+4%

**Source:** Nielsen Media Research, May 2011, 2013, 2016.

**Table 3:** Adolescent exposure to food and beverage television advertisements in May 2011, 2013 and 2016.

Food/Beverage Category	Average Number of Advertisements Viewed by Adolescents (Aged 12–17 Years)			
	May 2011 *n* (%)	May 2013 *n* (%)	May 2016 *n* (%)	% Change in Ad Exposure (*n*) May 2011 to 2016
Cakes	0.15 (<0.1)	-	0.66 (0.4)	+340%
Candy	3.40 (1.5)	9.62 (5.3)	9.96 (6.5)	+193%
Cereal	22.70 (10.3)	8.24 (4.6)	10.21 (6.6)	−55%
Cheese	17.18 (7.8)	10.24 (5.7)	10.58 (6.9)	−38%
Chocolate	22.55 (10.2)	21.54 (11.9)	7.62 (5.0)	−66%
Compartment snacks	-	0.67 (0.4)	-	-
Cookies	10.09 (4.6)	5.19 (2.9)	2.60 (1.7)	−74%
Ice cream	2.88 (1.3)	3.93 (2.2)	3.94 (2.6)	+37%
Pizza	6.24 (2.8)	2.76 (1.5)	1.31 (0.9)	−79%
Portable snacks	9.35 (4.2)	4.11 (2.3)	5.14 (3.3)	−45%
Snack foods	3.40 (1.5)	7.53 (4.2)	6.67 (4.3)	+96%
Fast food	74.76 (33.8)	68.00 (37.7)	64.05 (41.7)	−14%
Restaurants	12.50 (5.7)	10.83 (6.0)	8.14 (5.3)	−35%
Yogurt	19.19 (8.7)	10.31 (5.7)	5.06 (3.3)	−74%
Juices	10.10 (4.6)	10.20 (5.7)	8.36 (5.4)	−17%
Energy drinks	1.65 (0.8)	1.71 (0.9)	0.91 (0.6)	−45%
Soft drinks (regular)	2.69 (1.2)	5.36 (3.0)	5.67 (3.7)	+111%
Soft drinks (diet)	1.68 (0.8)	-	0.83 (0.5)	−51%
Sports drinks	0.75 (0.3)	0.25 (0.1)	1.91 (1.2)	+155%
TOTAL	221.26 (100)	180.49 (100)	153.62 (100)	−31%

**Source:** Nielsen Media Research, May 2011, 2013, 2016.

**Table 4:** Adolescent exposure to food and beverage television advertisements, in May 2011, 2013 and 2016, by food category and television station type.

Food/Beverage Category	Average Number of Advertisements Viewed by Adolescents (Aged 12–17 Years)											
	Children’s Specialty Stations (*n* = 2)				Teen Specialty Stations (*n* = 2)				Generalist Stations (*n* = 27)			
	May 2011	May 2013	May 2016	% Change May 2011 to 2016	May 2011	May 2013	May 2016	% Change May 2011 to 2016	May 2011	May 2013	May 2016	% Change May 2011 to 2016
Cakes	-	-	-	-	-	-	-	-	0.15	-	0.66	340%
Candy	2.04	4.35	7.89	+287%	0.29	0.45	1.67	476%	1.08	4.82	2.07	92%
Cereal	7.32	2.81	7.23	−1%	-	-	-	-	15.38	5.43	2.99	−81%
Cheese	4.74	2.76	4.06	-14%	0.36	-	-	-	12.08	7.47	6.52	−46%
Chocolate	0.87	1.72	1.60	+84%	1.01	0.33	0.14	−86%	20.66	19.49	5.87	−72%
Compartment snacks	-	-	-	-	-	-	-	-	-	0.67	-	
Cookies	1.71	0.55	0.17	−90%	0.82	0.25	-	-	7.56	4.39	2.43	−68%
Ice cream	0.43	1.56	0.63	+47%	-	-	-	-	2.45	2.36	3.31	35%
Pizza	0.23	-	0.39	+70%	-	-	-	-	6.01	2.76	0.92	−85%
Portable snacks	4.01	1.19	2.83	−29%	-	-	-	-	5.35	2.92	2.31	−57%
Snack foods	0.12	-	0.48	+300%	0.27	0.35	0.16	−41%	3.01	7.18	6.03	100%
Fast food	11.64	8.51	8.36	−28%	2.23	0.83	0.91	−59%	60.88	58.67	54.77	−10%
Restaurants	0.91	1.15	0.11	−88%	0.13	0.26	0.08	−38%	11.47	9.43	7.95	−31%
Yogurt	4.39	1.56	1.94	−56%	0.44	0.78	0.03	−93%	14.36	7.97	3.10	−78%
Juices	1.29	0.55	1.15	−11%	0.29	0.57	-	-	8.52	9.09	7.21	−15%
Energy drinks	-	0.18	0.06	∞	0.25	0.31	0.07	−72%	1.40	1.22	0.79	−44%
Soft drinks (regular)	0.39	0.70	0.51	+31%	0.60	1.24	0.26	−57%	1.71	3.41	4.89	186%
Soft drinks (diet)	-	-	-	-	-	-	-	-	1.68	-	0.83	−51%
Sports drinks	-	-	-	-	0.26	-	0.01	−96%	0.49	0.25	1.89	286%
TOTAL	40.09	27.58	37.41	−7%	6.94	5.36	1.67	−76%	174.24	147.54	114.54	−34%

**Notes:** Children’s specialty stations included Teletoon and YTV. Teen specialty stations included Much Music and MTV. All other stations were classified as generalist stations; Source: Nielsen Media Research, May 2011, 2013, 2016.

## Supplementary Materials

The following are available online at https://www.mdpi.com/2072-6643/12/2/428/s1, Table S1: List of television stations included in the study.
