# Supplementary material for: Exposure to Food and Beverage Advertising on Television among Canadian Adolescents, 2011 to 2016
Source: Nutrients. 2020 Feb 7;12(2):428. doi: 10.3390/nu12020428 (PMC7071192; doi:10.3390/nu12020428)
Supplement: Supplementary file 1 [file nutrients-12-00428-s001.pdf]

Table S1: List of television stations included in the study.

Television station			
**Nr.**	**Abbreviation**	**Name**	**Type**
1	**BRAV**	Bravo	Generalist
2	**CBLT**	CBC Television	Generalist
3	**CFTO**	CTV Television	Generalist
4	**CHCH**	CHCH	Generalist
5	**CITY**	City TV	Generalist
6	**CMT**	Country Music Television	Generalist
7	**COM**	The Comedy Network	Generalist
8	**CTV2**	CTV2	Generalist
9	**DISC**	Discovery Channel	Generalist
10	**FOOD**	Food Network	Generalist
11	**GLBL**	Global Television Network	Generalist
12	**HGTV**	HGTV Canada	Generalist
13	**HIST**	History Canada	Generalist
14	**M3 / MMM**	M3 (formerly MuchMoreMusic and MuchMore)	Generalist
15	**MTV**	MTV	Teen Specialty
16	**Much / MM**	Much (formerly MuchMusic)	Teen Specialty
17	**NWLD**	CBC News channel	Generalist
18	**OLN**	OLN (formerly Outdoor Life Network)	Generalist
19	**OMN1**	OMNI Television 1	Generalist
20	**OMN2**	OMNI Television 2	Generalist
21	**SCR**	The Score (2011, 2013)<br>Sportsnet360 (2016)	Generalist
22	**SHOW**	Showcase	Generalist
23	**SLIC**	Life Network (2011, 2013)<br>Slice (2016)	Generalist
24	**SPAC**	Space	Generalist
25	**SPNO**	Sportsnet Ontario	Generalist
26	**TROP / DTOR**	TVTropolis (2011, 2013)<br>DTour (2016)	Generalist
27	**TTE**	Teletoon English	Children's Specialty
28	**TWN**	The Weather Network	Generalist
29	**VIS**	Vision Television	Generalist
30	**W**	W Network (formerly Women's Television Network)	Generalist
31	**YTV**	YTV	Children's Specialty
